# Emerging Trends of Biomedical Signal Processing in Intelligent Emotion Recognition

**DOI:** 10.3390/brainsci14070628

**Published:** 2024-06-24

**Authors:** Ateke Goshvarpour

**Affiliations:** Department of Biomedical Engineering, Imam Reza International University, Mashhad 91735-553, Iran; ateke.goshvarpour@gmail.com

The field of biomedical signal processing has experienced significant advancements in recent years, particularly in the realm of emotion recognition. As society becomes increasingly aware of the significance of emotional intelligence and well-being, researchers are utilizing state-of-the-art technologies to develop innovative solutions for detecting and analyzing emotional states. This Special Issue explores the emerging trends in biomedical signal processing for intelligent emotion recognition and highlights the potential impact on our comprehension of human emotions.

The emerging trends in biomedical signal processing for intelligent emotion recognition have substantial implications for various fields, including mental health [1], neuroscience [2], robotics and human–machine interaction [3,4,5], advertising [6], and marketing [7]. Precise emotion recognition can aid in diagnosing and monitoring mental health conditions, such as depression and anxiety. Analyzing physiological signals can provide insights into the neural mechanisms underlying emotional processing. Intelligent emotion recognition can enhance human–machine interaction by enabling robots to respond to human emotions. Understanding consumer emotions can improve targeted marketing strategies and enhance customer satisfaction.

On the other hand, emerging trends in biomedical signal processing for intelligent emotion recognition hold significant promise for revolutionizing our understanding of human emotions. As researchers continue to push the boundaries of this field, we can expect to see innovative applications that transform various aspects of our lives. By leveraging cutting-edge technologies and interdisciplinary approaches, we can unlock the power of emotions and create a more empathetic and emotionally intelligent world. The past decade has witnessed a notable surge in research on EEG-based emotion recognition, with various methods and approaches being proposed to improve accuracy and reliability. This Special Issue brings together six cutting-edge articles that delve into the captivating world of emotion recognition using EEG signals and advanced artificial intelligence techniques. Specifically, it collects the latest advancements in EEG-based emotion recognition, from subject-independent emotion recognition to system-level reviews and future directions, highlighting the importance of understanding emotions and their neural correlates.

The first paper in this Special Issue, authored by Zhang et al., introduces a novel approach to subject-independent emotion recognition using a self-adaptive graph construction module. This model adapts by extracting EEG map features through the construction of multi-graphic layers to obtain a frequency band-based multi-graphic layer emotion representation. The authors demonstrate the effectiveness of their model on two public datasets, achieving superior performance compared to existing studies.

In the following paper, Yao et al. present a novel model that combines transformer and CNN architectures for EEG spatial–temporal feature learning. The proposed model utilizes position encoding and multi-head attention to perceive channel positions and timing information in EEG signals. Two parallel transformer encoders are used to extract spatial and temporal features from emotion-related EEG signals, and a CNN is used to aggregate the EEG’s spatial and temporal features, which are subsequently classified using Softmax. This innovative approach leverages the strengths of both models to extract complex spatial and temporal patterns from EEG signals, leading to improved accuracy in emotion classification tasks. By integrating transformer and CNN architectures, researchers can enhance the model’s ability to capture intricate relationships within EEG data, resulting in more robust emotion recognition systems. The authors demonstrate the effectiveness of their model on two public datasets, achieving high accuracy and outperforming state-of-the-art methods.

Goshvarpour and Goshvarpour propose a unique approach to emotion recognition using the Granger causality quantifier and EEG with combined electrodes. The necessity of evaluating large amounts of data for multi-channel measurements increases the computational cost of the EEG network. To date, several approaches have been presented to select the optimal cerebral channels, primarily relying on available data. As a result, the reduction in the number of channels has increased the risk of low data stability and reliability. Alternatively, this study suggests an electrode combination approach in which the brain is divided into six areas. By leveraging Granger causality analysis and combining EEG electrodes, the authors demonstrate the effectiveness of capturing complex EEG information for emotion recognition. This approach offers a new perspective for utilizing EEG signals to decode emotional states, highlighting the importance of spatial information in EEG signals.

The fourth article, authored by Yuvaraj et al., examines the application of a 3D-CNN with ensemble learning techniques for emotion recognition using spatio-temporal representation of EEG signals. The study explores hybrid models, incorporating decision layers such as multilayer perceptron, k-nearest neighbor, extreme learning machine, XGBoost, random forest, and support vector machine (SVM), alongside fully connected layers. Furthermore, the effects of post-processing and filtering output labels are investigated. By applying transfer learning with a pre-trained 3D-CNN MobileNet model on spatio-temporal representations of EEG signals from two public datasets, the authors demonstrate significant advancements in emotion classification tasks, surpassing previous methods. This research underscores the potential of merging advanced neural network architectures with ensemble learning strategies to improve the accuracy and resilience of emotion recognition systems.

In addition to these research papers, Al-Nafjan et al. present a systematic review research on neuro-tourism, shedding light on the application of neuroscience in the tourism industry. Neuro-tourism utilizes neuromarketing methods such as the brain–computer interface (BCI), eye tracking, galvanic skin response, etc., to develop tourism products and services that enhance the experience and satisfaction of tourists. This review highlights the potential of incorporating neuroscience principles into tourism practices to enhance visitor experiences and engagement. By leveraging insights from neuroscience, the tourism industry can create more personalized and immersive experiences for travelers, ultimately resulting in a more enriching and memorable journey. The authors suggest that the integration of artificial intelligence techniques is essential for advancing neuro-tourism research.

The final article, authored by Lim et al., provides a comprehensive review of EEG affective recognition from a neuroscience perspective. This review emphasizes the importance of integrating neural network models with neuroscientific evidence to enhance our understanding of emotional processing in the brain. The authors underscore the significance of comprehending the neural foundations of emotion recognition and discuss the potential benefits of biologically inspired modeling in advancing the field. By combining insights from neuroscience with advanced AI techniques, researchers can gain deeper insights into the neural mechanisms underlying emotions, paving the way for more sophisticated emotion recognition systems.

Overall, this Special Issue highlights the importance of harnessing advanced technologies and interdisciplinary approaches to enhance our understanding of emotions and their neural substrates. By combining insights from neuroscience, artificial intelligence, machine learning techniques, domain-specific knowledge, and psychology, researchers can develop more accurate and reliable emotion recognition systems that have the potential to transform various industries. We hope that the insights presented in these articles will inspire further research and innovation in the field of emotion recognition, ultimately leading to a more profound understanding of human emotions and their neural correlates.
